# Claremore Lake's Eutrophication: A Study of Happy Lake’s Input

**DOI:** 10.17912/micropub.biology.001742

**Published:** 2025-09-11

**Authors:** Cheyanne Olson, Ashli Yaqub, Dustin Browning

**Affiliations:** 1 Department of Natural Sciences, Northeastern State University, Tahlequah, Oklahoma, United States; 2 Department of Biology , Rogers State University, Claremore, Oklahoma, United States; 3 Grand River Dam Authority, Langley, Oklahoma, United States

## Abstract

Cultural eutrophication from human-driven nutrient loading threatens aquatic ecosystems globally. Claremore Lake, a drinking water source in Northeastern Oklahoma, is impaired by elevated chlorophyll-a. This study assessed nutrient inputs from Happy Lake, a retention pond connected to Claremore Lake. Monitoring from April through August of 2024 showed greater nutrient, oxygen, and algal fluctuations in Happy Lake than Claremore Lake. Results suggest targeted management strategies and further research on tributary sources like Happy Lake are needed to protect Claremore Lake’s water quality.

**Figure 1. Total Phosphorus By Sampling Location:**
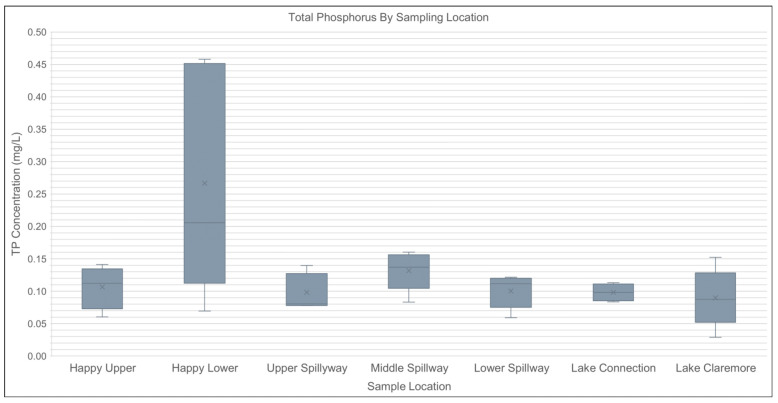
Total phosphorus (mg/L) measured between sample sites following flow from Happy Lake into Claremore Lake throughout the duration of the study. x represents the mean, a solid line represents the median, extended lines represent the maximum (above) and minimum (below) values for that site.

## Description

Cultural eutrophication is a global issue degrading freshwater ecosystems through the over-enrichment of nutrients, primarily nitrogen and phosphorus (U.S. EPA, 2024; Smith et al., 1999). These nutrients fuel the overgrowth of algae, increasing overall algal biomass and often leading to visible blooms that reduce water quality and disrupt aquatic communities (Dodds & Smith, 2016). While much attention has been given to harmful algal blooms dominated by toxin-producing cyanobacteria, non-toxic blooms can also create serious ecological problems. During algal respiration at night or decomposition events, dissolved oxygen (D.O.) can become depleted, stressing or killing aquatic organisms (Paerl et al., 2011). High algal biomass also reduces light penetration, suppressing photosynthesis in deeper water layers and altering both thermal and oxygen profiles (Thornton et al., 1990). In some systems, seasonal blooms of algae such as diatoms can cause taste and odor issues in drinking water supplies (Hilborn & Beasley, 2015; Hans & Pearl, 2008). Chlorophyll-a (chl-a), a proxy for algal biomass, increases with eutrophication and is monitored by the Oklahoma Department of Environmental Quality (ODEQ) to assess water quality. Exceeding state chl-a thresholds can indicate the potential for algal blooms (ODEQ, 2022b).

Claremore Lake is a 470-acre reservoir northeast of the City of Claremore in Rogers County, Oklahoma, with a conservation storage capacity of 7,900 acre-feet. Impounded in 1930 by the City of Claremore (Oklahoma Water Resources Board, 2010), Claremore Lake serves both recreational and municipal water supply needs. The lake’s 58-square-mile watershed is largely undeveloped and sparsely populated, with less than 1% classified as developed land (Oklahoma Water Resources Board, 2010). Its main tributaries, Dog Creek and Little Dog Creek, flow through predominantly pasture, grassland, and deciduous forest landscapes typical of the Central Irregular Plains (Woods et al., 2005). Claremore Lake is listed on Oklahoma’s 303(d) list of impaired waters due to elevated chlorophyll-a levels of unknown origin (Oklahoma Department of Environmental Quality, 2022a). Hydrologically connected to Claremore Lake, Happy Lake is a small retention pond that may function as both a nutrient sink and source, depending on hydrologic and biogeochemical conditions. Happy Lake has a dam height of 35 feet and a length of 320 feet, with a surface area of 6 acres and a storage capacity of 170 acre-feet. Preliminary observations indicate Happy Lake contains open water and areas of emergent and submerged aquatic vegetation, which influence nutrient cycling, oxygen dynamics, and algal growth. Understanding these processes and external nutrient inputs is essential for managing eutrophication in Claremore Lake.

The purpose of this study was to assess Happy Lake’s potential contribution to elevated chlorophyll-a levels in Claremore Lake. By measuring key water quality parameters at multiple sites between March and August 2024, this research aimed to improve understanding of nutrient dynamics and inform management strategies for eutrophication in Claremore Lake watershed.

Considerable variation was observed in water quality parameters across the seven sampling sites. Total phosphorus (TP) concentrations were highest at the Happy Lower site above the spillway (mean = 0.27 mg/L), while other sites remained closer to 0.10 mg/L. Mean total nitrogen (TN) concentrations ranged from 1.40 to 2.16 mg/L, with the highest concentrations also recorded at the Happy Lower between June-August. Algal particle counts varied widely, with the Lake Connection site exhibiting the greatest variability and highest mean count (mean = 7,860 particles; SD = 10,448), indicating substantial spatial heterogeneity in algal biomass.

Temperature showed both seasonal and spatial variation, with downstream sites in Claremore Lake exceeding 25°C during warmer periods. D.O. levels ranged from near hypoxic conditions (<1 mg/L) to over 10 mg/L. The lowest average D.O. concentrations were observed at the junction between Happy Lake and Claremore Lake, likely due to organic enrichment and microbial oxygen consumption. pH values varied across the system, with lower values (5.35–6.08) in the upstream Happy Lake sites (Sites 1–2) and more neutral to basic conditions (>7.0) at downstream locations in Claremore Lake (Sites 6–7).

A one-way ANOVA revealed a statistically significant difference in TP concentrations among sites (F₆ = 3.67, p = 0.01), suggesting localized inputs or nutrient cycling processes may be influencing phosphorus distribution, particularly near Happy Lake (Figure 1). Differences in TN (F₆ = 0.55, p = 0.77), temperature (F₆ = 0.59, p = 0.74), D.O. (F₆ = 0.81, p = 0.57), algal particle count (F₆ = 0.96, p = 0.48), and pH (F₆ = 1.02, p = 0.44) were not statistically significant across sites.

Several significant relationships among parameters were identified through Pearson correlation analysis. TN was positively correlated with TP (r = 0.72, N = 34, p < 0.01) and with water temperature (r = 0.68, N = 34, p < 0.01), suggesting a seasonal or thermal influence on nitrogen dynamics. Algal particle abundance showed a positive correlation with both TN (r = 0.41, N = 26, p = 0.04) and pH (r = 0.49, N = 34, p = 0.01), likely due to algal photosynthesis increasing pH during bloom events. D.O. was negatively correlated with TP (r = –0.40, N = 34, p = 0.02) and with water temperature (r = –0.49, N = 34, p < 0.01), consistent with the expectation that oxygen depletion occurs in warm, nutrient-enriched conditions. These patterns may reflect the development of algal blooms, followed by microbial decomposition of organic matter, which reduces oxygen availability. Critically, when D.O. drops below 2 mg/L, internal loading of phosphorus from sediments may occur, exacerbating eutrophic conditions through positive feedback.

The results of this study suggest that Happy Lake may contribute to elevated chlorophyll-a levels in Claremore Lake, particularly during spillover events or periods when water in the shallow spillover zone becomes stagnant and nutrient-rich, promoting algal growth. While total nitrogen and total phosphorus levels varied across sites; significantly higher TP concentrations were detected at the Happy Lake spillover (the connection where Happy Lake drains into Claremore Lake) suggesting localized enrichment (Nachtigall & Heim. 2023). Algal particle abundance also peaked at sites downstream of Happy Lake, with the highest variability and mean counts recorded within the middle of Claremore Lake, indicating the potential for episodic algal blooms driven by nutrient inputs. Statistical analyses further supported this link: TN was positively correlated with both TP and water temperature, while algal particles were significantly associated with higher TN and pH levels. These relationships suggest that warmer conditions and nutrient availability, particularly nitrogen, may be enhancing algal growth. The observed negative correlations between dissolved oxygen and both TP and temperature point to potential oxygen stress in nutrient-enriched areas - characteristic of eutrophic systems (Hou et al., 2013). Together, these findings highlight Happy Lake’s potential role as a nutrient and algal biomass contributor to Claremore Lake and underscore the need for targeted management strategies to monitor and mitigate nutrient inputs from this upstream source. Future studies should also direct focus on assessing input from Claremore Lake’s main tributaries, Dog Creek and Little Dog Creek.

## Methods

*Sample Collection and Preservation*

From March to August 2024, surface water samples were collected monthly at seven fixed sites spanning Happy Lake Reservoir, its spillway, and Lake Claremore. Site 1 was the easternmost, Happy Lake Upper, and Site 2 was Happy Lake Lower, located above the spillway where water flows out of Happy Lake. From there, water moves through the spillway gradient, Upper Spillway (Site 3), Middle Spillway (Site 4), and Lower Spillway (Site 5), before entering Claremore Lake at Site 6. Site 7, the westernmost site, was located further into Claremore Lake. Nutrient samples were collected in sterile 250 mL HDPE bottles and preserved with 2 mL of 5N sulfuric acid. Phytoplankton samples were stored in 50 mL HDPE centrifuge tubes and preserved with 0.5 mL glutaraldehyde for FlowCAM analysis (Vaulot et al., 1989). All samples were held at 4°C until analysis.

*In-situ Measurements*

At each site, pH, dissolved oxygen, and temperature were recorded using a Hydrolab Quanta probe. Additional 240 mL and 50 mL samples were collected for nutrients and algal biomass alongside particle counts, respectively, following standard protocols. Observations on turbidity, flow, pollution, fish kills, odors, and weather were also noted.

*Laboratory Analysis*

Samples were analyzed at the Grand River Dam Authority’s (GRDA) Water Quality Research Lab. Nutrients were digested using APHA Method 4500-P J (APHA, 2023) and analyzed via EPA Methods 127-C and 155-C on a SEAL AQ300 Discrete Analyzer. Phytoplankton samples were filtered (100 µm) and analyzed using a FlowCam Cyano (Yokogawa Fluid Imaging Technologies), which uses a 633 nm laser to image, count, and potentially classify algal particles.

*Data Analysis*

Statistical analyses, including ANOVA and Pearson correlation were performed using SPSS software and Microsoft Excel.
